# MSK1 regulates transcriptional induction of Arc/Arg3.1 in response to neurotrophins

**DOI:** 10.1002/2211-5463.12232

**Published:** 2017-05-11

**Authors:** Chris J. Hunter, Judit Remenyi, Sonia A. Correa, Lucia Privitera, Kathleen M. S. E. Reyskens, Kirsty J. Martin, Rachel Toth, Bruno G. Frenguelli, J. Simon C. Arthur

**Affiliations:** ^1^ MRC Protein Phosphorylation UnitCollege of Life SciencesSir James Black CentreUniversity of DundeeUK; ^2^ Wellcome Trust Centre for Gene Regulation and ExpressionWellcome Trust BuildingCollege of Life SciencesUniversity of DundeeUK; ^3^ Bradford School of PharmacyFaculty of Life SciencesUniversity of BradfordUK; ^4^ School of Life SciencesUniversity of WarwickCoventryUK; ^5^ Division of Cell Signalling and ImmunologyWellcome Trust BuildingCollege of Life SciencesUniversity of DundeeUK

**Keywords:** Arc/Arg3.1, BDNF, CREB, glutamate, histone H3, MSK1, neurotrophins, NMDA

## Abstract

The immediate early gene activity‐regulated cytoskeletal protein (Arc)/Arg3.1 and the neurotrophin brain‐derived neurotrophic factor (BDNF) play important roles in synaptic plasticity and learning and memory in the mammalian brain. However, the mechanisms by which BDNF regulates the expression of Arc/Arg3.1 are unclear. In this study, we show that BDNF acts via the ERK1/2 pathway to activate the nuclear kinase mitogen‐ and stress‐activated protein kinase 1 (MSK1). MSK1 then induces Arc/Arg3.1 expression via the phosphorylation of histone H3 at the Arc/Arg3.1 promoter. MSK1 can also phosphorylate the transcription factor cyclic‐AMP response element‐binding protein (CREB) on Ser133. However, this is not required for BDNF‐induced Arc.Arg3.1 transcription as a Ser133Ala knockin mutation had no effect on Arc/Arg3.1 induction. In parallel, ERK1/2 directly activates Arc/Arg3.1 mRNA transcription via at least one serum response element on the promoter, which bind a complex of the Serum Response Factor (SRF) and a Ternary Complex Factor (TCF).

**Abbreviations**
Arc: activity‐regulated cytoskeletal protein
BDNF: brain‐derived neurotrophic factor
CREB: cyclic‐AMP response element‐binding protein
CRE: cyclic‐AMP response elements
MSK1: mitogen‐ and stress‐activated protein kinase 1
NMDA: *N*‐Methyl‐d‐aspartic acid
SRE: serum response element

The transcriptional regulation of immediate early (IE) genes plays an important role in translating incoming signals into an appropriate cellular response. IE genes have been implicated in many neuronal processes ranging from development and synapse formation to synaptic plasticity and the encoding of memory 1, 2, 3. Multiple pathways have been implicated in the regulation of IE genes in neurons, however one pathway that has received significant attention is the classical MAPK pathway, which leads to the activation of ERK1 and 2 4. The ERK1/2 cascade can promote IE gene transcription via several mechanisms, including its ability to activate transcription from serum response elements (SREs) and cyclic‐AMP response elements (CREs) that are found in the promoters of many IE genes 2, 5.

Serum response elements can be bound by a combination of the serum response factor and an ETS domain transcription factor such as Elk1. ERK1 and 2 are able to directly phosphorylate Elk1, leading to transcriptional activation of SRE containing promoters 6. CREs are bound by cyclic‐AMP response element‐binding protein (CREB), or the related transcription factors ATF1 or CREM. Like Elk1, the transcriptional activity of CREB can be regulated by phosphorylation 7, 8. CREB was initially identified as a substrate of PKA downstream of cAMP signalling 7, 8. PKA‐mediated phosphorylation of CREB on Ser133 promotes CREB‐dependent transcription via the recruitment of the coactivator proteins CBP and p300 7. Subsequently, other kinases have been found to induce CREB Ser133 phosphorylation including mitogen‐ and stress‐activated protein kinase 1 (MSK1) and 2 downstream of the ERK1/2 and p38 MAPK pathways 9. The role of MSK‐mediated CREB phosphorylation was initially unclear, as it is unable to promote CBP or p300 recruitment 10. Analysis of CREB Ser133Ala knockin (KI) mice has however demonstrated that MAPK‐induced Ser133 phosphorylation of CREB is essential for the efficient induction of CREB‐dependent IE genes in fibroblasts and macrophages 11, 12.

Mitogen‐ and stress‐activated protein kinase localise to the nucleus and, in addition to CREB, they have been found to phosphorylate other nuclear proteins including the histone H3, HMG14 and Trim28 13, 14, 15. In line with this, a major function of MSKs is the regulation of a subset of IE genes, which has led to the discovery of roles for MSKs in the regulation of innate immunity 11, 16 and, as discussed below, neuronal function.

In most cell types, MSK1 and MSK2 are able to compensate for each other, and knockout of both MSK1 and MSK2 together is required to block the phosphorylation of MSK substrates 9, 17. In contrast, in primary cortical neuronal cultures, knockout of MSK1 alone is sufficient to prevent CREB phosphorylation in response to neurotrophins such as brain‐derived neurotrophic factor (BDNF), an effect that may be due to the low expression of MSK2 in neuronal cells 18. This importance for MSK1 in neurons is reflected in the finding that knockout of MSK1 in mice can result in behavioural deficits in a variety of models of learning and memory.

Mitogen‐ and stress‐activated protein kinase 1 is activated in hippocampal CA1 pyramidal neurons during fear conditioning 19 and MSK1 knockout mice have a mild memory formation deficit in Pavlovian fear conditioning and spatial learning models 20. In these experiments, MSK1 was suggested to be the major kinase responsible for both the CREB and histone H3 phosphorylation induced by these models 19, 20. In agreement with this, MSK1/2 double knockout mice showed a deficit in learning and histone phosphorylation in a forced swimming test, another paradigm of hippocampal memory 21. The role of MSK1 is not restricted to the hippocampus. Cocaine administration activates MSK1 in the striatum of mice, and MSK1 knockouts show decreased locomotor sensitisation in response to repeated cocaine administration 22, 23, 24. In addition, MSK1 has recently been implicated in the development of pain after injury 25, 26.

The mechanism underlying how MSKs affect CNS function is not fully understood. Loss of MSK1 protein or kinase activity does not appear to have any gross effects on CNS development 22, 27. MSK1/2 double knockout does however result in decreased spine density in the hippocampus and a lower proliferative rate in progenitor cells in the subgranular zone 28. These defects were especially apparent in mice that had been subject to a period of environmental enrichment (EE) 28 and may contribute to the lack of effect of enrichment on cognitive function in MSK1 knockout mice 29.

In agreement with this, enrichment enhanced the amplitude of miniature excitatory postsynaptic currents in wild‐type mice, but not mice with a D194A kinase dead KI mutation in the N‐terminal kinase domain of MSK1, which also showed a blunted increase in spine density following EE 27. MSK1 D194A KI mice raised under standard housing conditions, showed an impairment in basal synaptic transmission, but not long‐term potentiation, nor indeed several forms of spatial reference memory in the water maze 30. These observations suggest that the kinase activity of MSK1 may be required for longer term, homeostatic responses to changes in synaptic activity. This is consistent with the observation that MSK1 D194A KI hippocampal neurons fail to undergo homeostatic scaling in response to chronic (24 h) synaptic activity deprivation caused by blocking action potentials with the voltage‐gated sodium channel blocker TTX 27. Homeostatic scaling is a process that allows global regulation of synaptic strength within a neuron, and has previously been linked to both BDNF signalling 31 and the activity‐regulated cytoskeletal protein (Arc/Arg3.1), an IE gene widely implicated in a range of synaptic and experience‐dependent plasticity 32, 33, 34, 35. In wild‐type neurons, TTX treatment downregulated Arc/Arg3.1 protein levels, but this did not occur in MSK1 D194A KI neurons 27. How MSK1 regulates Arc/Arg3.1 was not however established in this study.

Here, we report that in primary cortical neuronal cultures, Arc/Arg3.1 transcription is directly regulated by MSK1 in response to BDNF, and that this occurs independently of CREB phosphorylation but correlates with the phosphorylation of histone H3 on the Arc/Arg3.1 promoter.

## Methods

### Mice

The generation and genotyping MSK1/2 double knockout, MSK1 D194A KI and CREB S133A KI mice has been described previously 17, 27, 36. MSK1 D194A KI mice were generated and maintained on a C57/Bl6 background. MSK1 and MSK2 knockout mice were backcrossed onto C57/Bl6 for 12 generations and the CREB S133A mice backcrossed to C57/Bl6 for six generations. As described in 36, the S133A mutation was induced in the endogenous CREB gene specifically in neurons using Nestin‐Cre; for these cultures CREB wild‐type Nestin‐Cre+ve cells were used as controls. All mice were maintained under specific pathogen‐free conditions in individually ventilated cages and allowed free access to food and water. Work was carried out under a UK Home Office license and approved by the University of Dundee and University of Warwick Welfare and Ethical Use of Animals Committee.

### Cortical neuronal culture

The neocortex was excised from postnatal pups (day 0 or 1), finely minced and digested 37 °C for 10 min with trypsin (0.05%, Invitrogen, Paisley, UK) in dissociation media (90 mm Na_2_SO_4_, 30 mm K_2_SO_4_, 16 mm MgCl_2_, 0.25 mm CaCl_2_, 32 mm HEPES). The tissue was rinsed by gravity precipitation twice in dissociation media and twice in Neurobasal A (Invitrogen) and gently triturated into a single‐cell suspension with a 5 mL plastic serological pipette. The supernatant containing single cells was spun down and the cells were resuspended in Neurobasal A (Invitrogen) supplemented with 2% B27, 1 mm l‐glutamine, 50 U·mL^−1^ penicillin, and 50 μg·mL^−1^ streptomycin (Invitrogen) and plated on poly‐d‐lysine‐coated 6 or 12 well plates (100 μg·mL^−1^; Sigma, Poole, UK). Cells were used between 7 and 10 days in culture at which point the density was 80–90% confluent.

Stimulation of cortical neurons involved application of BDNF (2, 10 and 50 ng·mL^−1^; Cambridge Bioscience, UK) and *N*‐Methyl‐d‐aspartic acid (NMDA; 20 μm; Sigma‐Aldrich). Enzyme and transcription inhibitors used in the study are as follows: the PI3 kinase inhibitor PI103 (10 μm; Merck, Watford, UK); the p38 inhibitor SB203580 (5 μm; Merck); the CaMK inhibitor Kn‐93 (10 μm; Merck); the MEK1/2 inhibitor PD184352 (2 μm; Axon Medchem, Groningen, Netherlands), the MSK1 inhibitor SB‐747651A (10 μm; Axon Medchem), the broad spectrum kinase inhibitor H89 (25 μm; Merck), and the inhibitor of transcription, actinomycin D (1 μg·mL^−1^; Sigma‐Aldrich).

### Immunoblotting

Cells were lysed directly into SDS sample buffer. Samples were run on 12% polyacrylamide gels and transferred onto nitrocellulose using standard techniques. Primary antibodies against ERK1/2, phospho ERK1/2, CREB and phospho‐Ser133 CREB were from Cell Signalling. The antibody against phospho‐Ser10 histone H3 was from Millipore. Detection was with HRP‐conjugated secondary antibodies (Pierce) using Supersignal ECL reagent (Pierce, Fisher Scientific ‐ UK Ltd, Loughborough, UK).

### qPCR

Following stimulation, total RNA was isolated using RNeasy kits (Qiagen, Manchester, UK) according to the manufacturer's protocols. RNA was reverse‐transcribed using iScript (Qiagen) and qPCR carried out using Sybergreen‐based detection (Bio‐Rad, Watford, UK or Takara, Saint‐Germain‐en‐Laye, France). The primers used are listed in Table 1. Fold‐stimulation was calculated relative to unstimulated wild‐type samples using 18s levels as a loading control 37.

**Table 1:** qPCR primer sequences

Use	Primer	Sequence
qPCR	Arc/Arg3.1 sense	CGAGCCCTGGTGTGGATAC
qPCR	Arc/Arg3.1 antisense	ACCGAGCCCTGCTTGAAC
qPCR	Nur77 sense	CCTGTTGCTAGAGTCTGCCTTC
qPCR	Nur77 antisense	CAATCCAATCACCAAAGCCACG
qPCR	18s sense	GTAACCCGTTGAACCCCATT
qPCR	18s antisense	CCATCCAATCGGTAGTAGCG
ChIP	P1	TGGTGTAAGTCAAGAAGG
ChIP	P2	AAGATGCCATATAAGGAATG
ChIP	P3	GTAATAACCTGCCTTAGCC
ChIP	P4	GTGACTAATGTGCTCTGC
ChIP	P5	CGAGCCCTGGTGTGGATAC
ChIP	P6	ACCGAGCCCTGCTTGAAC

### ChIP

Primary cortical neurons were either left unstimulated or stimulated with BDNF (50 ng·mL^−1^) for 30 min. Cells were then fixed for 10 minutes with 1% formaldehyde and chromatin was isolated using the chromatin immunoprecipitation (ChIP)‐IT Express magnetic kit (Active Motif, Carlsbad, CA, USA) according to the manufacturer's protocols. ChIP was performed using 6 μg phospho‐S10 Histone H3 antibody per IP. Primers sequences are provided in Table 1.

### Luciferase assays

To generate Arc/Arg3.1 reporter constructs, −1302 to +98 (sequence starting AGTGGGGGGCAT to CCTCCGGCACCG) of the Arc/Arg3.1 promoter was PCR amplified and cloned into the pGL4 luciferase vector (Promega, Southampton, UK). PCR‐based mutagenesis was used to mutate the SRE (CCTTATATGG to TTTTATATTT) and EGR‐binding site (CGGGCG to TTTTTT). All inserts were fully sequenced to ensure the absence of random PCR‐generated mutations. Cortical neurons were transfected at 3 DIV using Lipofectamine 2000 (Invitrogen) and analysed on day 5. A Renillia luciferase vector (pRL‐CMV, Promega) was used as a transfection control. Luciferase activity was detected in cell lysates using the dual luciferase assay system (Promega) according to the manufacturer's instructions.

### Statistical analysis

Data are presented as mean ± 1 SD of multiple biological replicates. Statistical analysis of the data involved one‐ or two‐way ANOVAs as appropriate as described in the figure legends. Statistical significance was accepted if *P* < 0.05. originpro 2016 (OriginLab, Northampton, UK) was used for statistical analysis.

## Results

### MSK1 promotes Arc/Arg3.1 transcription downstream of BDNF

**Figure 1:**
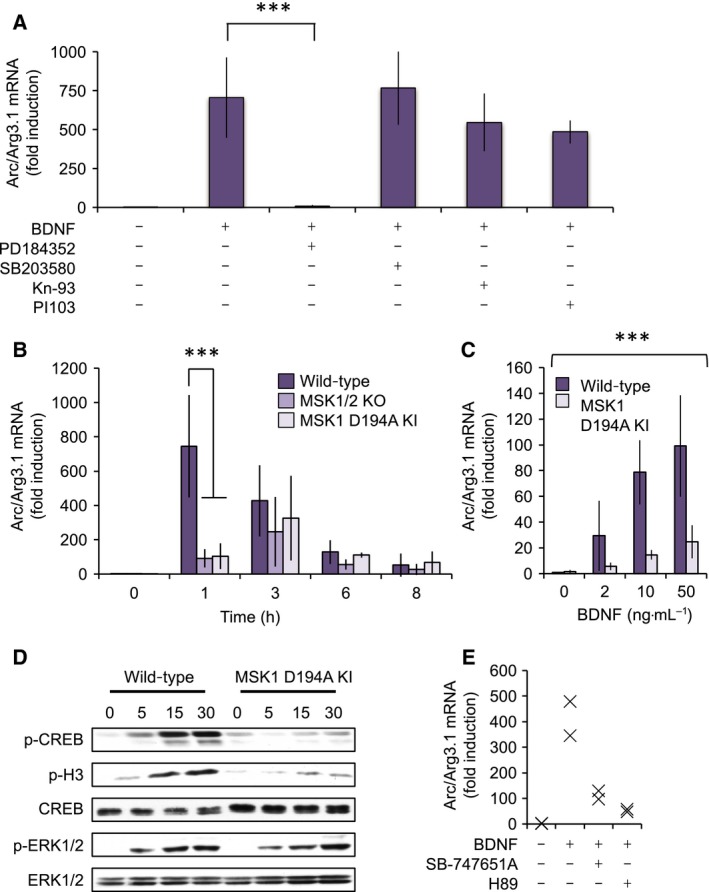
MSK1 promotes Arc/Arg3.1 mRNA induction downstream of BDNF. (A) Wild‐type cortical neuronal cultures were pretreated as indicated for 1 h with 2 μm PD184352, 5 μm SB203580, 10 μm Kn‐93 or 10 μm PI103. Cells were stimulated with 50 ng·mL^−1^ BDNF for 1 h, lysed and total RNA extracted. Induction of Arc/Arg3.1 mRNA was then determined by qPCR using 18s as a reference gene. Error bars represent the standard deviation of six stimulations except for PI103 where *n* = 3. A one‐way ANOVA showed a significant difference across the treatment groups (*F*_4,21_ = 14.802; *P* < 0.0001) with a selective inhibitory effect on BDNF‐induced Arc/Arg3.1 mRNA induction by PD184352, as assessed by a *post hoc* Bonferroni test (*P* < 0.0001, indicated by ***). (B) Cortical neuronal cultures were established from wild‐type, MSK1/2 double knockout (KO) or MSK1 D194A KI mice. Cells were stimulated with 50 ng·mL^−1^ BDNF for the indicated times and Arc/Arg3.1 mRNA induction determined by qPCR. Error bars represent the standard deviation of multiple experiments with 8–19 independent stimulations per condition. A two‐way ANOVA revealed a significant effect of genotype (*F*_2,148_ = 24.259; *P* < 0.0001) and duration of BDNF exposure (*F*_4,148_ = 32.446; *P* = 0), and a significant interaction between genotype and BDNF exposure (*F*_8,148_ = 14.559; *P* < 0.001) on Arc/Arg3.1 mRNA induction. *Post hoc* Bonferroni comparison showed a significant difference (*P* < 0.0001, indicated by ***) at 1 h between wild‐type and MSK1/2 KO, and wild‐type and MSK1 D194A KI, but no difference (*P* = 1) between MSK1/2 KO and MSK1 KI. There was no difference in Arc/Arg3.1 induction between genotypes at 3, 6 and 8 h. (C) Wild‐type or MSK1 D194A KI cortical neurons were stimulated with the indicated concentrations of BDNF for 60 min. The induction of Arc/Arg3.1 mRNA was then determined by qPCR. Error bars represent the standard deviation of eight stimulations per condition. A two‐way ANOVA showed a significant main effect of BDNF concentration (0–50 ng·mL^−1^) on Arc/Arg3.1 mRNA induction in wild‐type and MSK1 KI neurons (*F*_3,56_ = 31.136, *P* < 0.0001). Importantly, this induction also showed a main effect of genotype (*F*_1,56_ = 67.269, *P* < 0.0001) and an interaction between genotype and BDNF concentration (*F*_3,56_ = 12.697, *P* < 0.0001), indicative of a major role for MSK1 in the BDNF‐dependent induction of Arc/Arg3.1. A *P* value of < 0.0001 is indicated by ***. (D) Wild‐type or MSK1 D194A KI cortical neurons were stimulated with 50 ng·mL^−1^ BDNF for the indicated times and the levels of total and phospho ERK1/2, Ser10 phosphorylated histone H3 and total and Ser133 phosphorylated CREB determined by immunoblotting. (E) Wild‐type cortical neurons were pretreated as indicated for 1 h with either 10 μm SB‐747651A or 25 μm H89. Cells were then stimulated for 1 h with 50 ng·mL^−1^ BDNF and the induction of Arc/Arg3.1 mRNA determined by qPCR.

In cultures of primary cortical neurons, Arc/Arg3.1 mRNA levels were increased by stimulation with BDNF (Fig. 1). BDNF is known to activate a range of intracellular signalling pathways including PI3 kinase, Ca^2+^ and MAPK signalling pathways 38, 39. As kinase inhibitors are available that target these pathways 40, a range of compounds to inhibit the different BDNF‐activated signalling pathways was used. The induction of Arc/Arg3.1 mRNA following BDNF stimulation was not blocked by the PI3 kinase inhibitor PI103, the p38 inhibitor SB203580 or the CaMK inhibitor Kn‐93 (Fig. 1A) at concentrations previously established to inhibit the relevant kinases in cells 41. The induction of Arc/Arg3.1 mRNA was however completely abolished by PD184352 (Fig. 1A), a MEK1/2 inhibitor that blocks the activation of ERK1/2 41.

Although MSK1 can be activated by either the ERK1/2 or p38α MAPK pathways 42, we have previously shown in BDNF‐stimulated cortical neuronal cultures that the ERK1/2, but not the p38 pathway, is responsible for MSK1 activation 18. The requirement for ERK1/2 activation in Arc/Arg3.1 mRNA induction could therefore be consistent with a role for MSK1. Following stimulation with BDNF, in wild‐type cells the induction of Arc/Arg3.1 mRNA was maximal at 1 h, although strong induction was still seen at 3 h (Fig. 1B). Double knockout of MSK1 and 2 (MSK1/2 KO) greatly reduced the initial induction of Arc/Arg3.1 mRNA by BDNF at 1 h, although the knockout had less effect at 3, 6 and 8 h, where the induction of Arc/Arg3.1 was no different between wild‐type and MSK1/2 KO mice (Fig. 1B). In cortical neurons, MSK1 is expressed at much higher levels than MSK2 and knockout of MSK1 alone is sufficient to block BDNF‐induced CREB phosphorylation 18. To examine the importance of MSK1 kinase activity in the BDNF‐induced induction of Arc/Arg3.1 mRNA, we utilised an MSK1 KI mouse with a D194A mutation that destroys the catalytic activity of the N‐terminal domain 27. Although MSKs are unusual in containing two kinase domains, the only known role of the C‐terminal kinase domain is in the activation of the N‐terminal domain. Inactivation of the N‐terminal domain has previously been shown to block the phosphorylation of MSK substrates 43, 44. Analysis of Arc/Arg3.1 mRNA induction in neuronal cultures from MSK1 D194A KI mice gave similar results to MSK1/2 KO, with a significant attenuation of Arc/Arg3.1 mRNA induction by BDNF at 1 h (Fig. 1B). This difference between wild‐type and MSK1 D194A KI cells was also apparent when lower concentrations of BDNF (2 and 10 ng·mL^−1^) were used for stimulation (Fig. 1C). The absence of kinase activity in the MSK1 D194A mutant was confirmed by the reduction of CREB S133 and histone H3 S10 phosphorylation in response to BDNF in cortical neuronal cultures from the MSK1 D194A mice (Fig. 1D). To confirm the genetic results of MSK inhibition, we utilised a recently described MSK1 inhibitor, SB‐747651A 45. Pharmacological inhibition of MSK1 by SB‐747651A was able to reduce the induction of Arc/Arg3.1 mRNA by BDNF (Fig. 1E). Similar results were obtained with H89 (Fig. 1E), a compound that can target several kinases including MSK1 45.

### MSK1 regulates Arc/Arg3.1 transcription independently of CREB

**Figure 2:**
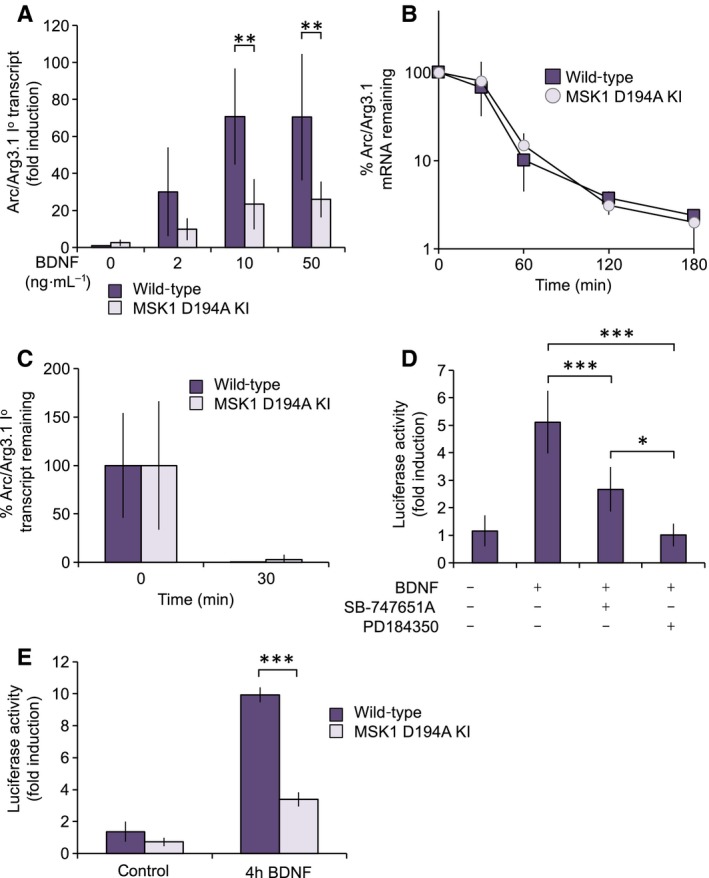
MSK1 regulates the induction of the Arc/Arg3.1 promoter. (A) Wild‐type or MSK1 D194A KI cortical neurons were stimulated with 2–50 ng·mL^−1^ BDNF for 60 min or left unstimulated. Induction of the primary transcript for Arc/Arg3.1 was determined by qPCR. Error bars represent the standard deviation of eight stimulations. A two‐way ANOVA showed a significant effect of treatment (*F*_3,56_ = 23.718; *P* < 0.0001) and genotype (*F*_1,56_ = 35.889; *P* < 0.0001), and a significant genotype × treatment interaction (*F*_3,56_ = 6.167; *P* = 0.0011). *Post hoc* Bonferroni comparison showed a significant difference in BDNF‐dependent Arc/Arg3.1 induction between wild‐type and MSK1 D194A KI neurons (*P* < 0.001) at 10 and 50 ng·mL^−1^ BDNF, with no difference in basal levels (*P* = 0.16) and with no significant stimulation by BDNF in MSK1 D194A KI neurons compared to control levels in MSK1 D194A KI neurons at any concentration of BDNF used. (B) Wild‐type or MSK1 D194A KI cortical neurons were stimulated with 50 ng·mL^−1^ BDNF for 90 min; 1 μg·mL^−1^ actinomycin D was added and mRNA isolated a 0, 30, 60, 120 and 180 min after addition. Arc/Arg3.1 mRNA levels were then determined by qPCR. Error bars represent the standard deviation of eight stimulations. A two‐way ANOVA showed no main effect of genotype on mRNA stability over the time period 0.5–3 h after BDNF induction (*F*_1,55_ = 0.504, *P* = 0.481), nor an interaction between genotype and time (*F*_3,55_ = 0.290, *P* = 0.832). (C) Wild‐type or MSK1 D194A KI cortical neurons were stimulated with 50 ng·mL^−1^ BDNF for 90 min; 1 μg·mL^−1^ actinomycin D was then added and mRNA isolated a 0 and 30 min after addition. Arc/Arg3.1 primary transcript levels were then determined by qPCR. Error bars represent the standard deviation of eight stimulations. (D) Wild‐type cortical neuronal cultures were transfected with an Arc/Arg3.1 luciferase reporter construct (corresponding to the promoter region 1302 to +98) as described in the Methods. Cells were pretreated as indicated for 1 h with 2 μm PD184352 or 10 μm SB‐747651A. Cells were then stimulated with 50 ng·mL^−1^ BDNF for 4 h, lysed and luciferase activity measured. Error bars represent that standard deviation of six transfections per condition. A one‐way ANOVA (*F*_3,20_ = 48.479; *P* < 0.0001) and subsequent *post hoc* Bonferroni comparison showed a significant elevation of luciferase activity by BDNF (*P* < 0.0001), which was attenuated by both SB‐747651A (*P* < 0.0001) and PD184350 (*P* < 0.0001). PD184350 was also more effective in attenuating BDNF‐stimulated luciferase activity than SB‐747651A (*P* = 0.044). (E) Wild‐type or MSK1 D194A KI cortical neurons were transfected with the Arc/Arg3.1 promoter luciferase reporter construct, stimulated with 50 ng·mL^−1^ BDNF for 4 h, lysed and luciferase activity measured. Error bars represent the standard deviation of measurements on independent cultures from three animals per genotype. A two‐way ANOVA showed a significant effect of treatment (*F*_1,8_ = 429.607; *P* < 0.0001) and genotype (*F*_1,8_ = 176.376; *P* < 0.0001), and a significant genotype × treatment interaction (*F*_1,8_ = 118.487; *P* < 0.0001). *Post hoc* Bonferroni comparison showed a significant difference in BDNF‐dependent luciferase induction between wild‐type and MSK1 D194A KI neurons (*P* < 0.0001). For A, D and E, *P* values of < 0.05, 001 and 0.0001 are indicated by *, ** and ***, respectively.

Changes in mRNA levels can reflect a change in either transcription or in mRNA stability. To address by which mechanism MSK1 might regulate Arc/Arg3.1 mRNA, the primary transcript for Arc/Arg3.1 was analysed and actinomycin D chase experiments carried out. Changes in the level of primary unspliced transcripts can give a better indication of changes in transcription compared to measurement of the spliced mRNA. In wild‐type cells, BDNF was able to increase the levels of the Arc/Arg3.1 primary transcript. This induction was significantly lower in MSK1 D194A KI cells (Fig. 2A). While this would suggest that MSK1 can regulate Arc/Arg3.1 transcription, it does not in itself exclude that MSK may also regulate Arc/Arg3.1 mRNA stability. To address this, actinomycin D was used to block transcription and the remaining Arc/Arg3.1 mRNA levels determined. In both wild‐type and MSK1 D194A KI cells, Arc/Arg3.1 was lost at a similar rate following actinomycin D addition (Fig. 2B). Analysis of the level of the primary unspliced Arc/Arg3.1 transcript in the actinomycin D chase experiment showed that it was greatly decreased with 30 min of actinomycin D addition (Fig. 2C), consistent with the rate of splicing being faster than the rate of degradation of the Arc/Arg3.1 mRNA. Together, the above results suggest that MSK1 regulates Arc/Arg3.1 mRNA induction primarily at the level of transcription.

To identify how MSK might regulate the Arc/Arg3.1 promoter, a 1.4 kb fragment corresponding to −1302 to +98 of the Arc/Arg3.1 genomic sequence was fused to a luciferase reporter and transfected into primary cortical neuronal cultures. This construct was inducible by BDNF, as evidenced by an increased luciferase activity following a 4 h treatment with BDNF (Fig. 2D). The induction of this reporter was reduced by either the ERK1/2 pathway inhibitor PD184352 or the MSK inhibitor SB‐747651A (Fig. 2D). The ability of BDNF to induce the luciferase reporter in MSK1 D194A KI cortical neuronal cultures was also lower than in wild‐type cells (Fig. 2E).

**Figure 3:**
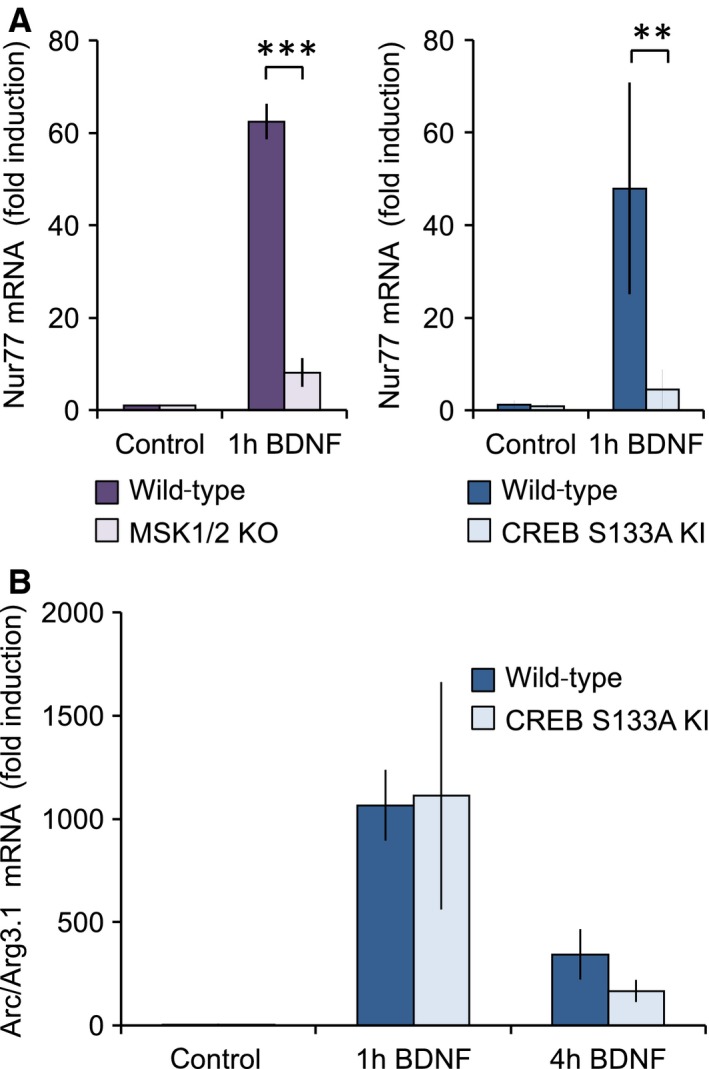
Arc/Arg3.1 mRNA induction by BDNF is independent of CREB phosphorylation. (A) Wild‐type, MSK1/2 knockout or CREB S133A KI cortical neuronal cultures were stimulated with 50 ng·mL^−1^ BDNF for 1 h and the induction of nur77 mRNA determined by qPCR. (B) Wild‐type or CREB S133A KI cortical neuronal cultures were stimulated with 50 ng·mL^−1^ BDNF for 1 or 4 h and Arc/Arg3.1 mRNA induction determined by qPCR. Error bars represent the standard deviation of four to six stimulations. For A, in the MSK1 KO experiments (left panel), a two‐way ANOVA showed a significant effect of BDNF treatment (*F*_1,12_ = 766.757; *P* < 0.0001) and genotype (*F*_1,12_ = 481.097; *P* < 0.0001), and a significant genotype × treatment interaction (*F*_1,12_ = 482.316; *P* < 0.0001) on BDNF‐dependent induction of Nur77. *Post hoc* Bonferroni comparison showed a significant difference in BDNF‐dependent luciferase induction between wild‐type and MSK1 KO neurons (*P* < 0.0001). In the CREB S133A KI experiments (right panel), a two‐way ANOVA showed a significant effect of BDNF treatment (*F*_1,13_ = 21.162; *P* < 0.001) and genotype (*F*_1,12_ = 15.868; *P* = 0.0016), and a significant genotype × treatment interaction (*F*_1,13_ = 15.484; *P* = 0.0017) on BDNF‐dependent induction of Nur77. *Post hoc* Bonferroni comparison showed a significant difference in BDNF‐dependent luciferase induction between wild‐type and CREB S133A KI neurons (*P* < 0.001), but no significant induction by BDNF of Nur77 in CREB S133A KI neurons. For B, a two‐way ANOVA revealed a significant effect of duration of BDNF exposure on Arc/Arg3.1 mRNA induction (*F*_2,29_ = 62.446; *P* < 0.0001), but no effect of genotype (*F*_1,29_ = 0.286; *P* = 0.597) and no interaction between genotype and duration of exposure (*F*_2,29_ = 0.684; *P* = 0.513) indicating that the induction of Arc/Arg3.1 mRNA is independent of the S133 residue of CREB. For A and B, *P* values of < 0.001 and 0.0001 are indicated by ** and ***, respectively.

To date, most of the target genes identified for MSKs are also regulated by the MSK substrate CREB 12. The majority of active CRE sites are thought to be located within 1 kb proximal to the transcriptional start site 46. *In silico* analysis of the sequence cloned into the Arc/Arg3.1 reporter did not reveal any strong consensus CREB binding sites within this region, suggesting that MSK1 may regulate Arc/Arg3.1 independently of CREB. It is possible however that CREB may bind outside the region used in the Arc/Arg3.1 reporter, and that the phosphorylation of CREB at these sites may contribute to the ability of MSKs to regulate Arc/Arg3.1 mRNA induction. To address this, we analysed the effect of a KI mutation of Ser133 to alanine in the endogenous CREB gene (CREB Ser133Ala KI 36). To confirm that the mutation of CREB at this site could affect BDNF‐induced transcription, we examined the induction of nur77, a known MSK and CREB target gene 12, 37. As expected, the induction of nur77 mRNA in response to BDNF was reduced by either MSK1/2 knockout or CREB Ser133Ala KI (Fig. 3A). The induction of the endogenous Arc/Arg3.1 mRNA by BDNF was however not reduced by the CREB Ser133Ala KI mutation (Fig. 3B).

### MSKs phosphorylate histone H3 at the Arc/Arg3.1 promoter

**Figure 4:**
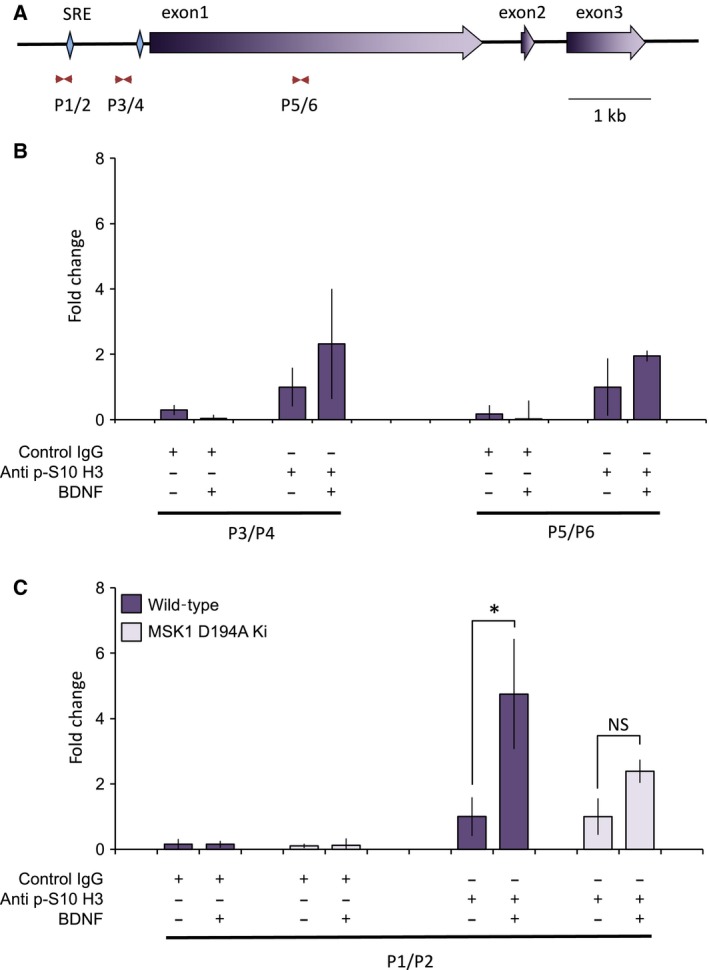
MSK1 promotes histone H3 phosphorylation at the Arc/Arg3.1 promoter. (A) The genomic structure of the Arc/Arg3.1 gene is shown including the positions of the SRE and Egr binding site. The positions of the three primer sets used for ChIP analysis are indicated. (B) Wild‐type primary cortical neurons were either left unstimulated or stimulated with 50 ng·mL^−1^ of BDNF for 30 min. Following cross‐linking, cells were lysed and samples prepared for ChIP prepared as described in the Methods. After ChIP using either a phospho‐Ser10 histone H3 antibody or control IgG, qPCR was used to quantify the levels of different regions of the Arc/Arg3.1 locus corresponding to the regions amplified by the p3/p4 or p5/p6 primer sets. Signal from input DNA was used to correct for loading, and fold change calculated relative to the unstimulated phospho Ser10 ChIP levels. (C) as (B) except primer P1 and p2 as well as cells from either wild‐type or MSK1 D194A KI neuronal cultures were used. Error bars represent the standard deviation of stimulations from three independent cultures. For the P3/P4 locus in B, a two‐way ANOVA showed a significant enrichment of the ChIP signal derived from the anti p‐S10 H3 antibody (*F*_1,8_ = 30.714; *P* < 0.001), but a *post hoc* Bonferroni comparison showed no significant effect of BDNF on the anti p‐S10 H3 signal (*P* = 0.0506). For the P5/P6 locus, a similar analysis revealed an enrichment of the ChIP signal by the anti p‐S10 H3 antibody (*F*_1,8_ = 14.730; *P* = 0.005), but no significant effect of BDNF on the anti p‐S10 H3 signal (*P* = 0.584). For the analysis of P1/P2 ChIP signals derived from the use of the anti p‐S10 H3 antibody in C, a two‐way ANOVA showed a significant effect of BDNF treatment (*F*_1,7_ = 17.778; *P* = 0.004), which a *post hoc* Bonferroni comparison showed was specific to wild‐type neurons (*P* = 0.012) since no significant stimulation was observed in neurons from MSK1 D194A KI mice (*P* = 1). For C, a *P* value of < 0.05 is indicated by *.

The above results indicate MSKs may regulate Arc/Arg3.1 induction independently of their ability to phosphorylate CREB. As MSKs also phosphorylate histone H3 on Ser10 13, the phosphorylation of histone H3 on the Arc/Arg3.1 promoter was examined by ChIP. As indicated in Fig. 4A, 3 primer sets were used to examine potential histone H3 phosphorylation at regions in both the Arc/Arg3.1 promoter and 1^st^ exon. In primary cortical neurons, BDNF strongly induced H3 phosphorylation in only one of these regions (Fig. 4B, C). The ability of BDNF to induce histone H3 Ser10 phosphorylation was significantly reduced in neurons isolated from the MSK1 D194A KI mice (Fig. 4C).

### Arc/Arg3.1 transcription is regulated by Srf sites

**Figure 5:**
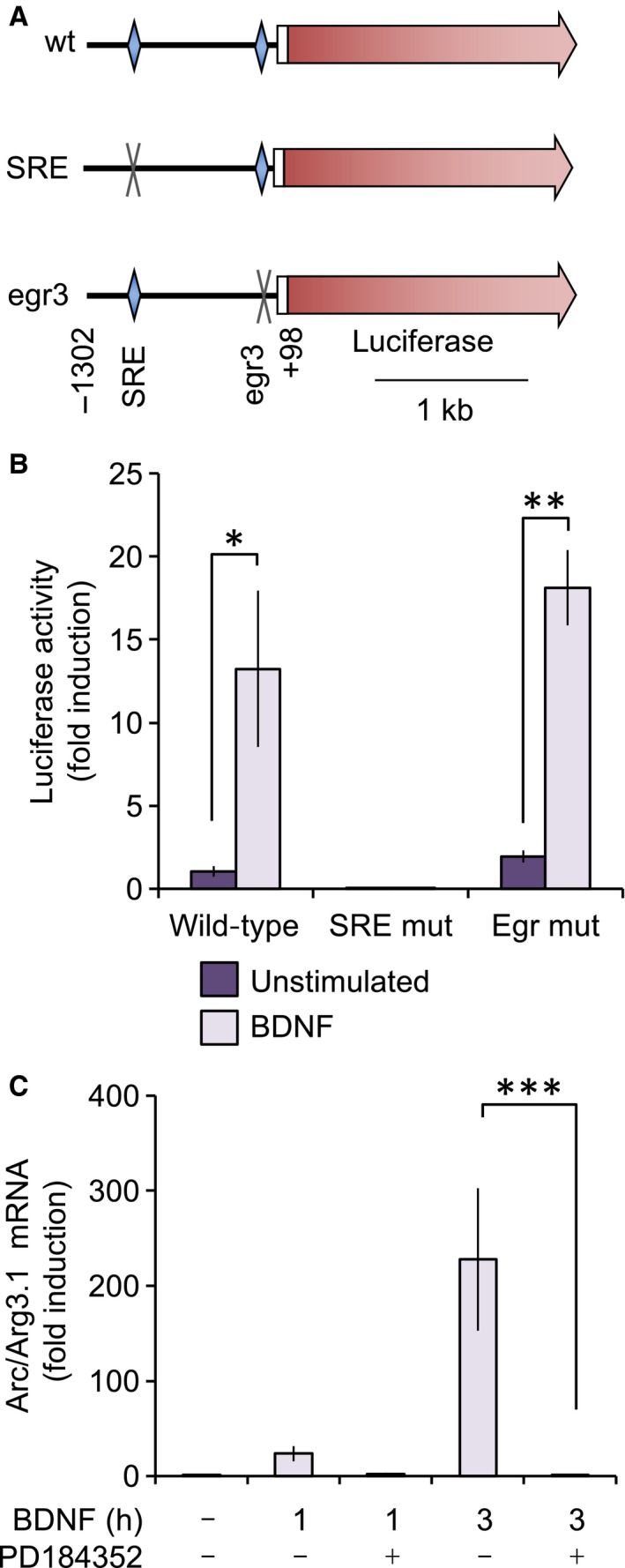
The Arc/Arg3.1 promoter is regulated by a SRE. (A) A −1302 to +98 fragment of the mouse Arc/Arg3.1 promoter was cloned into a luciferase reporter. Analysis of this sequence revealed the presence of a SRE and binding site for Egr transcription factors. Mutated versions of the reporter construct were created for each of these sites as described in the methods. (B) These constructs were transfected into wild‐type cortical neuronal cultures, which were either left untreated or stimulated for 4 h with 50 ng·mL^−1^ BDNF as indicated. Unpaired *t*‐tests revealed significant increases in luciferase activity in wild‐type and Egr mutant constructs compared to their corresponding unstimulated controls (*P* = 0.011 and *P* < 0.001, respectively), but no stimulation in the SRE mutant construct (*P* = 0.413). (C) MSK1 D194A KI cortical neuronal cultures were pretreated with 2 μm PD184352 for 30 min as indicated and were stimulated with 50 ng·mL^−1^ BDNF for the indicated times and Arc/Arg3.1 mRNA levels determined by qPCR. A one‐way ANOVA showed an overall significant difference across groups (*F*_4,13_ = 29.995; *P* < 0.0001) and *post hoc* Bonferroni comparison confirmed the effects of the MEK inhibitor as being highly significant at the 3 h time point (*P* < 0.0001). Error bars represent the standard deviation of between two and six independent stimulations. For B and C, *P* values of < 0.05, 0.001 and 0.0001 are indicated by *, ** and ***, respectively.

Both Srf and Egr transcription factors have been linked to Arc/Arg3.1 transcription 47, 48, 49. The regions analysed by ChIP are located next to a potential SRE and 5′ to an Egr family transcription factor binding site (Fig. 5A). To test if these sites were important for the BDNF activity at the Arc/Arg3.1 promoter, the effects of mutating these sites on the induction of the Arc/Arg3.1 promoter were examined. Loss of the Egr site did not prevent induction of the luciferase gene. However, mutation of the putative SRE site blocked both the basal and BDNF‐induced expression of the reporter (Fig. 5B). SREs bind a combination of Srf and a ternary complex factor such as Elk1. In response to mitogens and growth factors, ERK1/2 are able to phosphorylate Elk1, and thus help stimulate transcriptional activation from SREs 6, 50. ERK1/2 activation may therefore regulate the induction of Arc/Arg3.1 promoter both directly via phosphorylation of Elk1 and indirectly via the activation of MSK1. To test this, the effect of blocking ERK1/2 activation on the remaining Arc/Arg3.1 mRNA induction in response to BDNF in MSK1 D194A KI neurons was examined. In line with an additional MSK1‐independent role for ERK1/2, the remaining Arc/Arg3.1 mRNA induction in the MSK1/2 knockout cortical neurons was blocked by preincubation with the ERK1/2 pathway inhibitor PD184352 (Fig. 5C).

### MSK1 is not critical for the induction of Arc/Arg3.1 mRNA by NMDA

**Figure 6:**
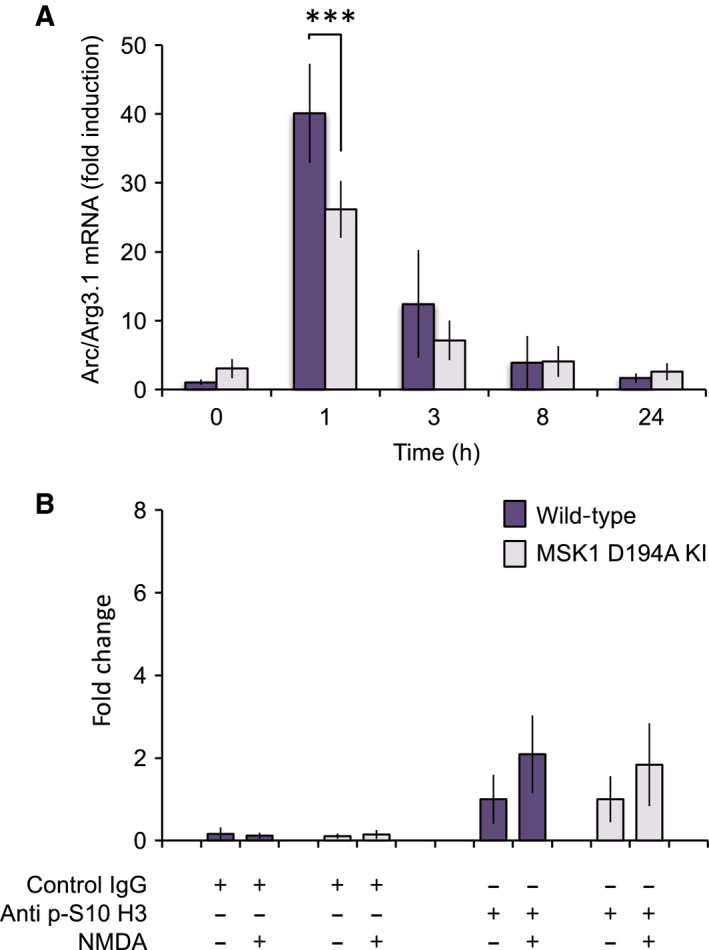
MSK1 activity is not critical to the induction of Arc/Arg3.1 mRNA by NMDA. (A) Wild‐type or MSK1 D194A KI neuronal cultures were stimulated for the indicated times with 20 μm NMDA. Cells were the lysed and Arc/Arg3.1 mRNA induction measured by qPCR. Error bars represent the standard deviation from eight stimulations per genotype. A two‐way ANOVA showed a significant main effect of genotype (*F*_1,69_ = 12.383, *P* < 0.001) on Arc/Arg3.1 induction by NMDA in wild‐type and MSK1 D194A KI neurons, which a Bonferroni *post hoc* comparison showed was significant at the 1 h time point (*P* < 0.0001). (B) Wild‐type or MSK1 D194A KI neuronal cultures were stimulated for 30 min with 20 μm NMDA. After cross‐linking, cells were lysed and samples prepared for ChIP prepared as described in the Methods. ChIP was carried out using either a phospho‐Ser10 histone H3 antibody or control IgG. qPCR was then used to quantify the change in histone phosphorylation at the Arc/Arg3.1 locus using the p1/p2 primer set shown in Figure 4A. For the analysis of ChIP signals derived from the use of the anti p‐S10 H3 antibody, a two‐way ANOVA showed no significant effects of NMDA treatment (*F*_1,7_ = 3.616; *P* = 0.099), nor of genotype (*F*_1,7_ = 0.063; *P* = 0.809) nor an interaction between the two (*F*_1,7_ = 0.063; *P* = 0.809). For A, a *P* value of < 0.0001 is indicated by ***.

In neurons, Arc/Arg3.1 mRNA has been shown to be induced by other stimuli in addition to neurotrophins. As MSK1 plays an important role in Arc/Arg3.1 induction downstream of BDNF, we also examined if it played a similar role in response to the glutamate receptor agonist NMDA. As expected, Arc/Arg3.1 mRNA levels were induced by NMDA in wild‐type cells (Fig. 6A). Compared to what was observed with BDNF, the MSK1 D194A KI neurons had only a relatively small effect on Arc/Arg3.1 mRNA induction in response to NMDA stimulation (Fig. 6A) indicative of a positive role for MSK1 in the induction of Arc/Arg3.1 following the activation of NMDA receptors. NMDA was less effective at promoting the induction of Arc/Arg3.1 mRNA (compare fold induction between Figs 1A and 6A). A stronger induction of Arc/Arg3.1 mRNA by BDNF relative to NMDA has also been reported in rat cortical neurons 51. Consistent with the lower induction and reduced dependency on MSK1, NMDA did not strongly induce the phosphorylation of histone H3 at the Arc/Arg3.1 promoter in the region of the SRE (Fig. 6B).

## Discussion

We show here that MSK1 is required for the maximal induction of Arc/Arg3.1 mRNA in response to BDNF in primary neuronal cultures. This was independent of the ability of MSKs to phosphorylate CREB but correlated with the phosphorylation of histone H3 at the Arc/Arg3.1 promoter. While most MSK‐regulated genes described to date are regulated by CREB phosphorylation 9, the ability of MSK to phosphorylate other substrates suggests that CREB‐independent MSK‐mediated gene regulation will occur. Related to this, recent studies have proposed that MSKs may also regulate gene induction at the level of chromatin via phosphorylation of histone H3 52, 53, 54. While many of the stimuli that induce CREB activation also stimulate Arc/Arg3.1 expression, a clear link from CREB to the Arc/Arg3.1 promoter has not hitherto been established.

Arc/Arg3.1 has been found to be upregulated following intrahippocampal infusion of BDNF in an ERK1/2‐dependent manner. This study also showed that CREB Ser133 phosphorylation was also ERK1/2‐dependent in this model 55. Previous reports have however noted the lack of a CREB binding site in the proximal Arc/Arg3.1 promoter 56. A CREB binding site has, however, been identified in a synaptic activity‐responsive element 5 kb upstream of the Arc/Arg3.1 gene 57. The binding of CREB to this element was demonstrated by ChIP and mutation of the CREB binding site in this element reduced its ability to respond to increased neuronal activity in reporter assays in cultures from the rat neocortex or hippocampus 57. In contrast, in a mouse cortical neuron culture system, we did not observe any difference in Arc/Arg3.1 induction in response to BDNF in neurons expressing a Ser133Ala CREB mutation. In wild‐type cells, BDNF was able to stimulate CREB phosphorylation in the cortical neuronal cultures (Fig. 1D) suggesting that this was not due to a failure to activate CREB. Consistent with this induction of mRNA for nur77, a known CREB target was reduced in the CREB S133A KI neurons. The difference between our findings and those in 57 could be explained by several factors. Synaptic activity and BDNF may utilise different transcription factors and promoter elements to control Arc/Arg3.1 expression 58. Alternatively, the activity of CREB at the synaptic activity‐responsive element in the Arc/Arg3.1 promoter may be independent of Ser133 phosphorylation. For example, CRTC coactivator proteins can bind to CREB independently of Ser133 phosphorylation 59, 60. Furthermore, it was recently shown that CRTC3 recruitment can promote CREB‐dependent transcription in cells from CREB Ser133Ala KI mice demonstrating that in some circumstances CREB function can be Ser133‐independent 11.

The synaptic activity‐responsive element also contained binding sites for the serum response complex and MEF2, and this MEF2–CREB–SRE motif has subsequently been identified in other activity‐regulated genes 61. The identification of SREs in Arc/Arg3.1 promoter has been reported previously 57, 62. These studies, which were also performed in primary neuronal cultures, have shown that in addition to the proximal SRE shown in Fig. 4, at least two more distal SREs exist in the Arc/Arg3.1 promoter 57, 62. In particular, a distal SRE 6.5 kb upstream of Arc/Arg3.1 has been identified as being important to the maximal induction of Arc/Arg3.1 by BDNF 62. Knockout of the serum response factor has also been found to reduce Arc/Arg3.1 induction following mGlu5 stimulation of striatal neurons or in the dentate gyrus following electroconvulsive shock 48, 49. In the present study, we find that the proximal SRE in the Arc/Arg3.1 promoter can drive the expression of a luciferase reporter. Furthermore, we find that histone H3 phosphorylation is induced by BDNF in this region via the ERK1/2 activated kinase MSK1, and that this, as opposed to the phosphorylation of CREB S133, is associated with the induction of Arc/Arg3.1. Of note, while the proximal promoter is sufficient to allow induction of the Arc/Arg3.1 mRNA, our results do not exclude the possibility that the distal SRE sites are involved and may also be regulated in an MSK‐dependent manner.

The role of histone H3 phosphorylation in the direct regulation of Arc/Arg3.1 expression has not been extensively studied. However, changes in chromatin structure 63 or histone post‐translational modifications in cells have been found to correlate with changes in Arc/Arg3.1 expression in response to a variety of *in vitro* and *in vivo* stimuli e.g. 25, 26, 64. Perhaps the most direct are observations that a complex comprising the histone demethylase PHF8 and the acetyltransferase TIP60 regulate the activity‐dependent induction of Arc/Arg3.1 in a manner that is correlated with the acetylation of lysine 9 and phosphorylation of serine 10 on histone H3 (H3K9acS10P) 65; knockdown of PHF8 resulted in both a reduction in Arc/Arg3.1 expression and H3K9acS10P. However, no mechanism was provided for the phosphorylation of H3 Ser10, leaving open the possibility that MSK1 may be involved, especially since histone H3 acetylation and phosphorylation occur *in vivo* in an MSK1‐dependent manner 20, 21.

The phosphorylation of histone H3 by MSK1 would require the targeting of MSK1 to chromatin, but the mechanism by which this occurs is not fully understood. Our data could agree with a model in which the SRE may be recruiting MSK1 to this region of chromatin. A similar mechanism has been proposed to occur in HeLa cells at SREs in the c‐fos and egr1 promoters where knockdown of the ternary complex factor Elk1 reduced MSK1 recruitment to these promoters 66. Further studies would be required to determine in this mechanism operated at either the proximal or distal SREs in the Arc/Arg3.1 promoter. In particular, while our studies indicate that MSKs may be able to regulate the proximal region in the Arc/Arg3.1 promoter, they do not exclude that MSKs may also play a role at the distal control elements. Equally, it will be important to establish the general nature of these observations beyond primary neuronal cultures and in the developmentally mature central nervous system.

In conclusion, our observations are consistent with a prominent role of MSK1 in BDNF‐dependent regulation of Arc/Arg3.1, an IE gene critical for a range of synaptic and experience‐dependent synaptic plasticity 35. Moreover, our findings of the importance of histone H3 phosphorylation in Arc/Arg3.1 induction provide a novel mechanism of Arc/Arg3.1 regulation involving epigenetic modifications of the genome and chromatin remodelling. Such modifications are increasingly becoming appreciated as prime regulators of synaptic and cognitive function and as therapeutic targets for a range of neurological disorders 67, 68. Given the ability of MSK1 to regulate Arc/Arg3.1 induction, MSK1 represents a plausible target for drug discovery programs aimed at improving cognition.

## Author contributions

Carried out experiments and analysed data: CJH, SAC, LP, KMSER, KM, RT; wrote paper and directed study: BGF, JSCA.
